# Introducing human papillomavirus (HPV) primary testing in the age of HPV vaccination: projected impact on colposcopy services in Wales

**DOI:** 10.1111/1471-0528.16610

**Published:** 2020-12-15

**Authors:** F Pesola, M Rebolj, S Leeson, L Dunk, L Pickford, A Gjini, P Sasieni

**Affiliations:** ^1^ Cancer Prevention Group School of Cancer & Pharmaceutical Sciences Faculty of Medicine and Life Sciences King’s College London London UK; ^2^ Department of Obstetrics and Gynaecology Betsi Cadwaladr University Health Board Bangor UK; ^3^ Public Health Wales Cardiff UK

**Keywords:** Cervical screening, colposcopy, human papillomavirus, human papillomavirus vaccination, workload

## Abstract

**Objective:**

To determine the demand for colposcopy in the Cervical Screening Wales programme after the introduction of human papillomavirus (HPV) cervical screening, which coincided with the start of screening of women vaccinated against HPV types 16/18.

**Design:**

The study used a computational model that assigns screening and screening‐related colposcopy events to birth cohorts in individual calendar years.

**Setting:**

Cervical Screening Wales.

**Population:**

Women aged 25–64 years from birth cohorts 1953–2007.

**Methods and main outcome measures:**

We estimated the numbers of colposcopies and high‐grade cervical intraepithelial lesions (CIN2+) within Cervical Screening Wales in 2018–32, using official population projections for Wales and published estimates of the effects of HPV screening and vaccination.

**Results:**

Vaccination will reduce the number of colposcopies by 10% within the first 3–4 years after the national roll‐out of HPV screening, and by about 20% thereafter. The number of screening colposcopies is estimated to increase from 6100 in 2018 and peak at 8000 (+31%) in 2021, assuming current screening intervals are maintained. The numbers of CIN2+ lesions follow similar patterns, stabilising at around 1000 diagnoses per year by 2026, approximately 60% lower than at present. Extending the screening intervals to 5 years for all women shows similar trends but introduces peaks and troughs over the years.

**Conclusions:**

Vaccination will not fully prevent an increase in colposcopies and detected CIN2+ lesions during the first 2–3 years of HPV‐based screening but the numbers are expected to decrease substantially after 5–6 years.

**Tweetable abstract:**

HPV‐based cervical screening will initially increase colposcopy referral. In 6 years, this increase will be reversed, partly by HPV vaccination.

## Tweetable abstract

HPV‐based cervical screening will initially increase colposcopy referral. In 6 years, this increase will be reversed, partly by HPV vaccination.

## Introduction

High‐risk human papillomavirus (HPV) testing is more sensitive for the detection of high‐grade cervical intraepithelial neoplasia (CIN2+), and is now replacing liquid‐based cytology (LBC) as the primary test in cervical screening. Earlier studies estimated that, despite careful selection through triage testing, this will result in a large initial increase in the demand for colposcopy.^1^

The size of the increase in colposcopies has not been carefully modelled and is complicated by the introduction of HPV‐based screening having coincided with the beginning of screening of birth cohorts that were partially vaccinated against the most oncogenic HPV genotypes, 16 and 18. Vaccination lowers the need for colposcopy by reducing the risk of persistent HPV infections and cervical abnormalities.^2^ How this reduction will balance the increase expected on account of HPV testing cannot be directly observed from earlier screening studies,^1^ because they included predominantly unvaccinated women.

We estimated the impact over time (to 2032) of the introduction of HPV screening on colposcopy services including detection of CIN2+ in Wales, given that the youngest cohorts now entering the screening programme have been offered vaccination against HPV. This includes women who were vaccinated either aged 14–18 years or, from September 2020 onwards, those who were vaccinated aged 12–13 years.

## Methods

In Wales, girls aged 12–13 years have been offered HPV vaccination since September 2008. A catch‐up campaign was run for girls aged 14–18 years in 2008–2011 (see Supplementary material, Table S1). Cervical Screening Wales offers screening to women aged 25–49 years every 3 years and to those aged 50–64 years every 5 years.^3^ Following a local pilot launched in April 2017 (unpublished data from Public Health Wales), Wales switched from LBC with reflex HPV testing to primary HPV screening with reflex LBC in September 2018.^4^ The recommended clinical management of abnormalities for both screening tests is presented in Table 1.

**Table 1:** Clinical management of women screened under LBC and HPV protocols, as recommended by the UK National Screening Committee

	LBC and HPV triage	HPV and LBC triage
Baseline	Cyt –ve: routine recall HPV –ve low‐grade* cyt: routine recall Other cyt +ve:** colposcopy	HPV ‒ve: routine recall HPV +ve & cyt +ve: colposcopy HPV +ve & cyt –ve: early recall (12 months)
12‐month early recall		HPV ‒ve: routine recall HPV +ve & cyt +ve: colposcopy HPV +ve & cyt –ve: early recall (24 months)
24‐month early recall		HPV –ve: routine recall HPV +ve: colposcopy

cyt, cytology; HPV, human papillomavirus; LBC, liquid‐based cytology; ‒ve, negative; +ve, positive.

* Approximately equal to atypical squamous cells of undetermined significance (ASCUS) and low‐grade squamous intraepithelial lesions (LSIL) in the Bethesda 2014 classification.

** Women with high‐grade cytological abnormalities regardless of the HPV status, and women with borderline or low‐grade cytological abnormalities combined with a positive HPV test.

Although this is likely to change in the future, there are at present no plans to reduce screening intensity for cohorts with high vaccination coverage.

The number of women aged 25–64 years alive between 2018 and 2032 (birth cohorts 1953–2007) was retrieved from age‐specific population projections from the Welsh Office for National Statistics.^5^ We accounted for women who will have undergone a hysterectomy while they belong to the screening target age group, using English rates by 5‐year age group.^6^

**Figure 1:**
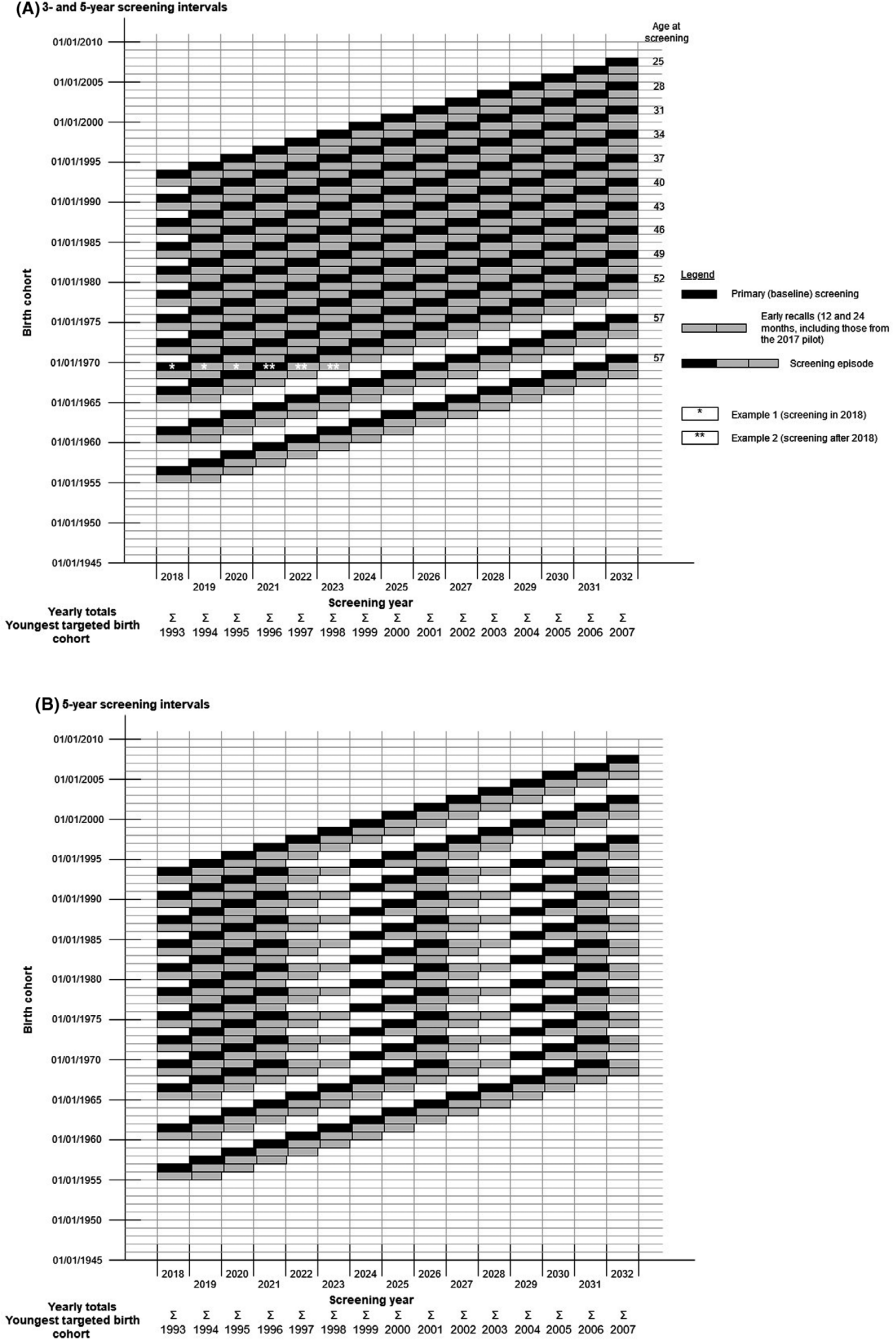
Screening episodes in the calculations. (A) Scenario with 3‐yearly routine recall intervals for women screened below age 50 yearas, and with 5‐yearly intervals for older women. (B) Scenario with 5‐yearly routine recall intervals for all women regardless of age, effective after the first HPV‐based screen (Appendix).

We calculated the outcomes after 2018 for two main screening scenarios. In the first (Figure 1A), women were invited at 3‐ and 5‐year intervals, i.e. at ages 25, 28, 31, 34, 37, 40, 43, 46, 49, 52, 57 and 62. In the second (Figure 1B), women entering the screening programme at age 25 in 2019 or later would be invited at 5‐year intervals as recommended by the UK National Screening Committee,^7^ i.e. at ages 25, 30, 35, 40, 45, 50, 55 and 60 years. Women aged <50 years who had entered the screening programme before 2019 would receive their first invitation for HPV testing 3 years after their last cytology screen and would thereafter follow age‐independent 5‐year intervals. To account for women who are not regularly screened, we assumed that 10% of screens in the subsequent screening rounds are from women attending their first HPV‐based screen.

Twenty percent of the targeted women were included in the Welsh pilot between April and December 2017 (three‐quarters of the calendar year). In 2018, 54% of the invited women were screened with HPV testing: 20% of women invited between January and May, 50% of those who were invited between June and August (they were included in the extended pilot), and 100% after the national roll‐out in September. To calculate the totals, these two different screening pathways were combined using a weighted sum of screening‐related colposcopies and CIN2+ diagnoses for HPV‐based and LBC‐based screenings; for example, for 2017, the following formula was applied: 0.75*(0.2*HPV pathway + 0.8*LBC pathway) + 0.25*1*LBC pathway. Calendar year 2018 was used as the reference baseline.

The 3.5‐year screening coverage at age 25–49 years, and the 5‐year coverage at age 50–64 years in Wales in 2017/18 were 70.8% and 73.7%, respectively (3‐year coverage for women 25–49 years of age is not routinely reported). Assuming that the coverage rates will remain stable, we applied them in all screening rounds.^4^ For simplicity, we assumed that all women who undergo screening participate in the invitation year, although they may miss an invitation and attend following their next invitation 3 or 5 years later.

Calculations are explained in Figure 1. Women with positive screening tests were assumed to be managed as described in Table 1. Parameter values for screening outcomes in unvaccinated women are listed in Table 2. The numbers of detected CIN2+ were calculated as a proxy for the number of CIN treatments (this is most likely an overestimate, acknowledging that a proportion of women with CIN2 do not opt for treatment). Age‐specific outcomes were not reported from the Welsh pilot. The overall screening positivity for both HPV testing and cytology were, however, similar in the Welsh and the English pilots. Hence, we used published age‐specific data from the English pilot, where screening outcomes were reported for unvaccinated cohorts screened in 2013 or 2014, and followed up until mid‐2017.^8^ Age‐specific CIN2+ detection was estimated in our model through the positive predictive value (PPV) of a screening‐related colposcopy at baseline and after the two early recalls (at 12 and 24 months). This was done separately for the first (prevalence) and the subsequent (incidence) rounds of HPV‐based screening. Screening‐related colposcopies and CIN2+ were attributed to the same calendar year as the screening or early recall tests that resulted in the colposcopy referral. The total yearly numbers were then obtained by summing across birth cohorts.

**Table 2:** Parameters for screening outcomes in unvaccinated women used in the calculations, by screening test and screening round. Proportions of the screening population, unless otherwise indicated

	Setting	Total	25–29 years	30–49 years	50–64 years
Baseline colposcopy referral^a^	Cytology screening	3.8%	9.6%	3.0%	1.2%
	HPV screening – first round	4.2%	10.8%	3.2%	1.4%
	HPV screening – subsequent rounds	1.5%^a^	3.1%^e^	1.2%^e^	0.6%^e^
Adherence to colposcopy at baseline (if referred)^a^	All	95%	95%	95%	95%
Attended colposcopy after ER12 + ER24^b^	HPV screening – first round	2.8%	6.3%	2.3%	1.4%
Proportion of ER12 + ER24 colposcopies that were due to ER12^c^	HPV screening – first round	44%	44%	44%	44%
Attended colposcopy at ER12^f^	HPV screening – subsequent rounds	0.4%	0.8%	0.3%	0.2%
Attended colposcopy at ER24^f^	HPV screening – subsequent rounds	0.5%	1.0%	0.4%	0.2%
PPV for CIN2+ at baseline colposcopy	Cytology screening	42%^a^	50%^d^	37%^d^	25%^d^
	HPV screening – first round	41%^c^	49%^d^	37%^d^	25%^d^
	HPV screening – subsequent rounds^g^	33%	39%	30%	20%
PPV for CIN2+ at ER12	HPV screening – first round	35%^c^	42%^d^	32%^d^	21%^d^
	HPV screening – subsequent rounds^g^	29%	34%	25%	17%
PPV for CIN2+ at ER24	HPV screening – first round	13%^c^	16%^d^	12%^d^	8%^d^
	HPV screening – subsequent rounds^g^	11%	12%	9%	6%

CIN, cervical intraepithelial neoplasia; cyt, cytology; ER, early recall (the number describes the recommended number of months after the baseline screen); HPV, human papillomavirus; PPV, positive predictive value.

^a^ As reported from the English pilot.^8^

^b^ Calculated as [% with colposcopy at any time^8^ – (% referred to colposcopy at baseline × % adherence to colposcopy)].

^c^ Calculated from estimates for a triage protocol not including HPV 16/18 genotyping for cytology‐negative women in the English pilot.^19^

^d^ An average colposcopy at age 24–29 years was associated with an approximately 20% higher PPV for CIN2+ than an average colposcopy for all ages combined. At age 30–49 years, the PPV for an average colposcopy was about 10%, and at age 50–64 years it was about 40% lower than an average colposcopy for all ages combined. These values were consistent for HPV testing and cytology as primary screening tests.^8^

^e^ At baseline screening in the subsequent HPV round of the English pilot, HPV positivity was about half of that observed at baseline screening in the first HPV round.^8^ We assumed that this relationship was constant across the entire age span, so that an estimated 14.0% at age 24–29, 5.2% at age 30–49 and 2.8% at age 50‐64 years tested HPV positive in the subsequent round. As observed in this pilot,^8^ we further assumed that 22% of women who tested positive for HPV in the subsequent round were referred to colposcopy at baseline, again the assumption was that this relationship was constant across all ages. However, those observations were based on the English pilot’s 3‐year interval. For a 5‐year interval in women below 50 years of age, we finally estimated the proportions referred to colposcopy by applying a factor of 5/3.^23^

^f^ We previously estimated based on the English pilot that, without using HPV 16/18 genotyping for triage of cytology negative women, an additional 27% of colposcopies are made after the 12‐month early recall (compared with the number of colposcopies made at baseline) and an additional 33% colposcopies (again compared with the number of colposcopies made at baseline) are made after the 24‐month early recall.^19^ The values were observed for the first HPV round and we assumed that a similar relationship would be observed for the subsequent HPV rounds. To move from a 3‐year to a 5‐year interval in women screened below 50 years of age, we applied a factor of 5/3 as explained above.

^g^ Values were reduced by 20% compared to the first HPV round, in accordance with the observations for CIN3+ detection from the Dutch POBASCAM randomised trial.^11^

We calculated the numbers of women with a colposcopy or a detected CIN2+. The published programme statistics indicate that 5271 women were referred for colposcopy from the screening programme between April 2017 and March 2018.^4^ On average, roughly 2800 women had been referred each screening year for other (clinical) reasons. The available official statistics indicate that, on average, each woman referred through the screening programme undergoes 1.3 colposcopies, whereas women referred for other reasons undergo between 1.4 and 1.5 colposcopies. We did not attempt to estimate the numbers of women with CIN2+ diagnosed through non‐screening colposcopies, but they are rare (fewer than 150 per year).^4^

The proportion of women in Wales vaccinated against HPV was reported in the national vaccination statistics by school‐year (see Supplementary material, Table S1).^9^ The cohorts in our screening scenarios included women who were vaccinated as part of the catch‐up programme with the bivalent vaccine (Cervarix; GlaxoSmithKline, Brentford, UK), or as part of the routine programme with either bivalent or, since September 2012, quadrivalent vaccine (Gardasil; Merck, Kenilworth, NJ, USA). We modelled vaccination coverage of 55% among girls targeted for catch‐up vaccination, and of 85% among girls targeted for routine vaccination. Both estimates are approximate averages across the relevant school‐year cohorts.^9^

For the vaccinated cohorts, screening parameters were estimated by adjusting those for unvaccinated cohorts (Table 2) for vaccine effectiveness (see Supplementary material, Appendix S1). After adjustment for vaccine coverage rates, the assumed population‐based vaccine effectiveness in reducing the need for screening‐related colposcopies was 14% among the catch‐up cohorts, and 34% and 26% among routine cohorts vaccinated with the bivalent and the quadrivalent vaccines, respectively. We applied these estimates to colposcopies at baseline and at early recalls. Similarly, the vaccine effectiveness in reducing the overall rates of CIN2+ was estimated at 19% in the catch‐up cohort, and 58% and 44% in the routinely vaccinated cohorts with bivalent and quadrivalent vaccines, respectively.

We tested the robustness of our predictions by varying the following screening and vaccination outcome parameters (see Supplementary material, Table S2). In the subsequent HPV screening rounds, we halved and doubled the proportion of screens (i.e. to 5% and 20%, respectively, from the base‐case value of 10%) that are made in women attending their first HPV‐based round (to capture women who are irregularly screened). In the English pilot, baseline colposcopy referrals in the first round of screening with HPV testing varied between the six participating laboratories within the range of +/‒ one‐third around the mean observed for the entire pilot.^10^ In the sensitivity analysis, we decreased and increased all input screening‐related colposcopy parameters by one‐third. Referrals in the first subsequent HPV round of the English pilot have only been reported for the 3‐year interval in women who screened negative and were <50 years of age in the first HPV screening round. Eventually, the subsequent HPV screening rounds will include also women who had HPV infections in the first round but who were referred to routine recall after viral clearance at 12 or 24 months. Women with cleared infections have a higher cumulative risk of abnormalities than women who were HPV negative at screening.^11^ In a sensitivity analysis, therefore, we assumed that the proportion of women with a screening‐related colposcopy and those with CIN2+ in the first complete subsequent HPV round was 50% higher than has so far been reported for HPV‐negative women. An increase of this magnitude would be slightly greater than that observed in the Dutch POBASCAM trial, which suggested an approximately 30–40% increment. This was inferred on the basis that 3.4% of women in that trial had a positive HPV test with negative cytology,^12^ and that, in the subsequent HPV round, the risk of a CIN2+ lesion among HPV‐positive, cytology‐negative women who cleared the infection by the first repeat test was about ten times higher than in HPV‐negative, cytology‐negative women.^11^ Uncertainty also remains around the PPV of HPV testing for CIN2+ during the subsequent HPV screening rounds. In the sensitivity analyses, we assumed both that the PPV remains the same as in the first HPV round, and that it decreases by half. For vaccination, we ran additional analyses by increasing the effect of vaccination for herd immunity, and, separately, to the level observed in Scotland in women screened at age 20.^13^ In the Scottish data, a significant herd immunity effect was observed only in the routinely vaccinated population.^8^, ^13^ In unvaccinated women from routinely vaccinated birth cohorts, herd immunity was considered to have reduced the number of CIN2 cases by 67%. We assumed that this estimate was representative for the numbers of colposcopies and CIN2+ cases. Finally, we repeated the analysis under the condition of no vaccination effect, which is equivalent to not implementing a vaccination programme.

Patient or public representatives were not involved in the design or the conduct of this study. A relevant core outcome set is not available yet in the ‘Core Outcomes in Women’s and Newborn Health’ (CROWN) database and, therefore, was not used in this study.

The study was funded by Cancer Research UK (grant numbers: C8162/A25356, C8162/A27047) and Public Health Wales.

## Results

**Figure 2:**
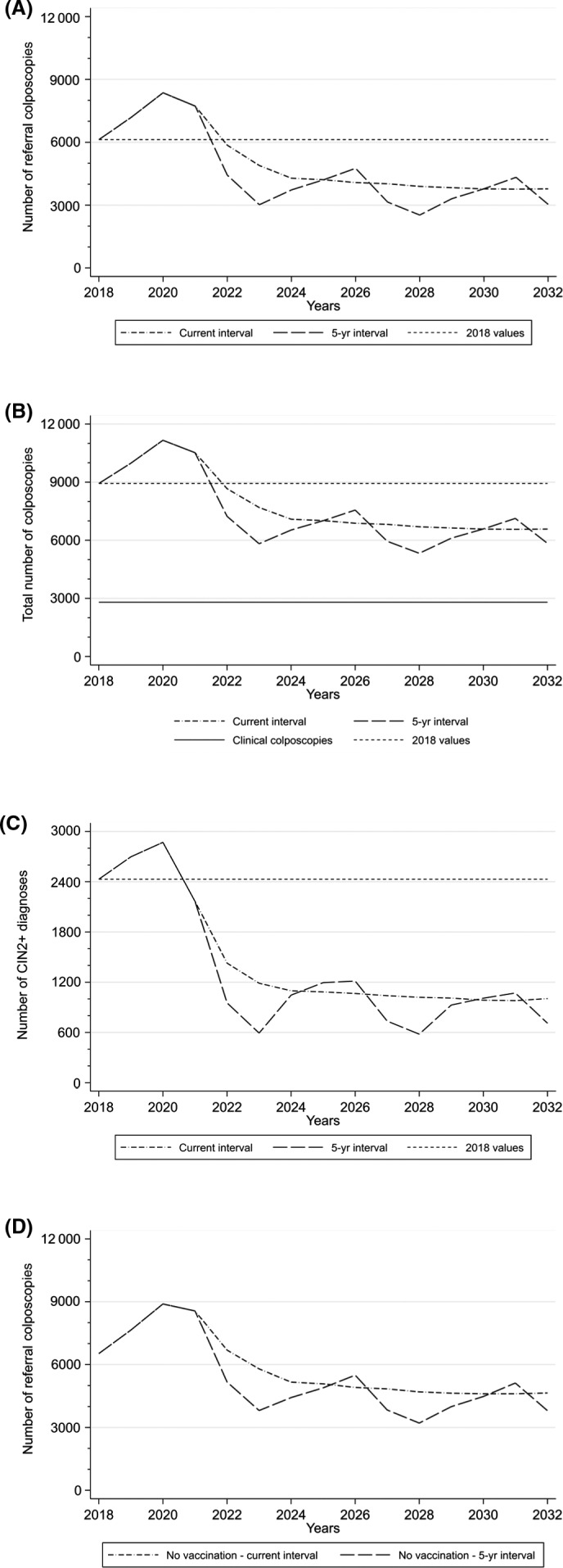
Results from the main analysis. (A) Numbers of women undergoing a screening‐related colposcopy. (B) Total numbers of women undergoing colposcopy (i.e. screening‐related or clinical colposcopy). (C) Numbers of women with a CIN2+ diagnosis after a screening referral, by screening scenario. (D) Numbers of women undergoing a screening‐related colposcopy estimated under the no vaccination scenario.

Had there been no vaccination, the yearly number of women referred to colposcopy after a non‐negative screening test would peak around 9000 in 2020/21, and thereafter stabilise around 5000 per year by 2026, at first about 10%, and then about 20% higher than in the situation with vaccination (Figure 2, Figure S1A, and higher‐resolution Figure 2, Figures , S3–S6). With vaccination, and assuming that screening intervals remain the same as they are at present (i.e. 3 years below, and 5 years above age 50), our projections show that the number of women with a screening‐related colposcopy would rise from around 6100 in 2018 to a peak of about 8000 (+31%) in 2020/21 (Figure 2B). Thereafter, the numbers are projected to fall, first to 6000 in 2022 (−2% compared with 2018), and should then gradually stabilise around 3800–4000 by 2026 (‒34% compared with 2018 and ‒50% compared with the peak in 2021).

Including colposcopies made for other reasons, the total numbers of women with a colposcopy would increase from 9000 in 2018 to 11 000 in 2020 and 2021 (+23%), and stabilise around 7000 (−22% compared with 2018) by 2026 (Figure 2A). Acknowledging that some women undergo more than one colposcopy, the estimated total numbers of colposcopy appointments are close to 12 000 in 2018, between 14 000 and 15 000 at the peak in 2020–2021, and 9000 in a stable situation by 2026.

Switching to a 5‐year screening interval for all women would still produce the same peak for screening‐related colposcopies within the first 3 years of HPV testing (Figure 2). However, a longer interval for young women, in whom most colposcopies are made, now produces a larger drop by 2022 (to 4400 women with a screening‐related colposcopy, or ‒28% compared with 2018) and 2023 (to 3000, or ‒50% compared with 2018). This would be followed by a series of 5‐yearly peaks and troughs which would oscillate, roughly, between 3000 and 4500 per year as explained above.

The projections for the number of women with CIN2+ follow similar patterns (Figure 2C). From about 2400 women with CIN2+ in 2018, the number is expected to rise to 2900 in 2020 (+21%). Thereafter, the number is expected to drop steadily, for example, to 1200 in 2023 (‒50% compared with 2018), 1100 in 2025 (‒54% compared with 2018), and to stabilise at around 1000 by 2026 (‒58% compared with 2018). As with colposcopies, the number of CIN2+ is also expected to oscillate around the estimated number of 1000 per year with the introduction of a uniform 5‐year screening interval.

All sensitivity analyses confirmed a gradual increase in the numbers of women undergoing a screening‐related colposcopy (see Supplementary material, Figure S1A) and with a detected CIN2+ lesion (Figure S2A) by 2022, and a subsequent sharp decrease followed by a gradual stabilisation; peaks and troughs also remain visible with an extended 5‐year interval (Figures S1B and S2B). Most of the analyses predict almost 4000 women with a screening‐related colposcopy and close to 1000 of those with a CIN2+ diagnosis per year after stabilisation. Across all studied years, the values were most affected when we varied the proportions of women referred to a screening‐related colposcopy by +/– one‐third around the base‐case value observed in the English pilot. The numbers of CIN2+ are predicted to be 15–20% lower than in the base‐case analysis when assuming a higher effectiveness of the vaccine based on Scottish data and 5–10% lower when accounting for herd immunity. Under both sensitivity analysis scenarios suggesting a stronger vaccination effect, the differences with the base‐case analysis increased towards the later years as more vaccinated women have entered the screening programme.

## Discussion

### Main findings

In countries such as Wales, the national roll‐out of HPV testing has coincided with the first birth cohorts vaccinated against HPV 16/18 reaching the screening eligibility criteria. Our projections are that Cervical Screening Wales will see important fluctuations in the demand for colposcopy and CIN2+ diagnostics. Within the first 3 years of HPV‐based screening, the yearly numbers of women with screening‐related colposcopies are projected to increase by about a third (from 6100 to 8000). This is due to the first screen with a sensitive HPV test. In subsequent years, the numbers are expected to gradually stabilise at 3800–4000, taking into account the uncertainty in several input parameters. This means that after the peak in 2020/21, the demand for screening‐related colposcopy could be around 30–40% lower compared with the current numbers. With an introduction of a longer screening interval, the numbers would continue to fluctuate around this level until 2032 (and possibly beyond). The numbers of women with a CIN2+ diagnosis will follow similar patterns, stabilising at levels that are 50–60% below those recorded at present.

### Strengths and limitations

We note that our calculations did not allow for variation in the timing of screening and early recall appointments (e.g. due to missed appointments or pregnancies), and we assumed that screening behaviour will not change over time. In terms of the effect of vaccination, our estimates could be considered conservative. We defined women as vaccinated if they received a full recommended course with either three (for girls born until 2000/01) or two doses (for girls born in 2001/02 and thereafter). A significant level of protection against CIN2+ from a single dose has not yet been demonstrated in the UK context.^13^ Ideally, data for all screening parameters would derive from the Welsh setting but had to be substituted by those from England where the overall screening test positivity appeared to be similar. Nevertheless, the English pilot used a range of HPV assays (including APTIMA as used in Wales) and that could skew the results. The assay used in Wales tends to be more specific for high‐grade CIN.^14^ Finally, a margin of error should be acknowledged for the absolute numbers in our base‐case results, although these calculations managed to satisfactorily reproduce several reported parameters. For example, the calculated number of screening‐related colposcopies in 2017 was similar to the officially reported numbers (6000 versus 5300, or +13%), and the increase in the detection of CIN2+ with HPV testing versus LBC during the entire screening episode was 40% (non‐tabulated) for unvaccinated birth cohorts (compared with a previously reported estimate of 49%).^8^

### Interpretation

The sharp increase in the number of early recall colposcopies appears to be confined to the first few years of HPV‐based screening. These years coincide with the very early days of population‐based screening of cohorts vaccinated through the catch‐up campaign. Hence, at the population level, vaccination is initially expected to prevent only about 10% of screening‐related colposcopies. The increase would require additional colposcopy capacity, with the prospect that this additional capacity would not be needed within 10 years (this is consistent with data from the randomised trials).^15^, ^16^ Careful planning is needed to ensure that extra colposcopy clinics are booked and that staff (nurses and gynaecologists) workload is appropriately reassigned. One solution could be to increase the specificity of the primary screening test, e.g. by using HPV assays that are more specific, like the APTIMA mRNA assay,^17^, ^18^ which was chosen by Cervical Screening Wales. Nevertheless, even assays such as APTIMA can still lead to very high positivity among young women.^19^ In the Welsh pilot, more than 20% of women screened at age 25–29 years had HPV detected on APTIMA (unpublished data from Public Health Wales). Although triage biomarkers such as HPV genotyping are proving to be popular in some countries, they could further increase the need for colposcopy during the already challenging early HPV screening period.^20^ Biomarkers that would improve the specificity of triage to colposcopy without a loss in screening sensitivity are needed, but none have yet been sufficiently validated.^21^ Other options could include strategies that reduce the referral at 24 months (when the PPV is the lowest), for example by delaying colposcopies in women with persistently negative cytology; or a re‐definition of the cytology threshold for an immediate referral, for example an increase to high‐grade cytological abnormalities. Almost a third of all colposcopies in Wales in 2017/18 were made for reasons other than the screening programme.^4^ Restriction of non‐screening‐related colposcopy referrals, for example by requiring an abnormal HPV test or a strong suspicion on a clinical examination, could be considered. Similar restrictions could be considered for colposcopies after a failure of test‐of‐cure.

Our projections are consistent with those previously reported from Australia,^22^ and show that the change from a 3‐year to a 5‐year interval will have a long‐lasting effect on colposcopy referrals, as well. The number of referrals will oscillate for years to come and could alternate between halving and doubling within a span of a few years. It is unrealistic to expect for the screening programme to be able to cope with such unsustainable peaks and troughs without any changes in the organisation. Therefore, there is a need to optimise the roll‐out of a longer interval in a way that would make the screening workload more constant and manageable over the years. While optimisation of workloads is beyond the scope of this analysis, we will be reporting on this in the future.

Our estimates will form the basis for a more realistic colposcopy and CIN2+ diagnostics workload planning in Cervical Screening Wales. Although the calculations were informed by the best available data from either Wales or from countries with similar screening programmes, the results are informative also for other cervical screening programmes. Another, more methodological, insight derived from this analysis is relevant for future evaluations of HPV screening. Arguably, one of the aims of such evaluations will be to demonstrate a high detection of CIN2+. Often, routine services are evaluated by a comparison with historical controls, which in this case would be screened with cytology or would include a lower penetration of vaccinated cohorts among the women eligible for HPV‐based screening. With our calculations, we showed that the outcomes of HPV‐based screening will need to be interpreted with a robust understanding of the vaccination effect. This is because at the population level, the effect of vaccination on colposcopies and CIN2+ will manifest itself gradually. It will increase over time, thereby obscuring the effect of HPV screening alone.

## Conclusions

During the first 3 years of a national roll‐out of HPV testing in Cervical Screening Wales, which coincides with screening of the first partially vaccinated birth cohorts, the yearly screening‐related colposcopy referrals will increase by about a third. Nonetheless, 5–6 years after roll‐out these numbers will decrease to be approximately 40% lower than the current ones. By 2026, the number of CIN2+ should stabilise at a level that is 60% lower than at present.

### Disclosure of interests

MR reports a speaker fee from Hologic paid to employer. PS is a statistician on a trial where Merck is providing HPV vaccine for free. FP, SL, LD, LP and AG declare no conflict of interest. Completed disclosure of interests forms are available to view online as supporting information. The Public Health Wales Screening Laboratory that processes and reports cervical screening samples for Cervical Screening Wales currently has a contract with Hologic for the provision of equipment and consumables for HPV testing and liquid‐based cytology.

### Contribution to authorship

FP developed the study concept, conducted the analyses, drafted the paper and made critical revisions. MR and PS developed the study concept and made critical revisions. SL. LD. LP and AG made critical revisions. All authors made the decision to submit.

### Details of ethics approval

Approval by ethics committee was not sought as all data were previously published.

### Funding

The study was funded by Cancer Research UK (grant numbers: C8162/A25356, C8162/A27047), and partly funded by Public Health Wales. FP was funded by Cancer Research UK (grant number: C8162/A25356), and PS and MR by Cancer Research UK (grant number: C8162/A27047).

## Supporting information


**Figure S1**. Sensitivity analyses for the number of women undergoing colposcopies under the current screening interval (A) and 5‐yearly intervals (B).

**Figure S2**. Sensitivity analyses for the number of women with a CIN2+ diagnosis, under the current screening interval (A) and 5‐yearly intervals (B).

**Figure S3**. Panel A of Figure 2: numbers of women undergoing a screening‐related colposcopy.

**Figure S4**. Panel B of Figure 2: total numbers of women undergoing colposcopy (i.e. screening‐related or clinical colposcopy).

**Figure S5**. Panel C of Figure 2: numbers of women with a CIN2+ diagnosis after a screening referral, by screening scenario.

**Figure S6**. Panel D of Figure 2: number of women undergoing a screening‐related colposcopy estimated under the no vaccination scenario.

**Table S1**. HPV vaccination coverage in Wales, by birth cohort. Data reported in COVER reports.
**Table S2**. List of parameters used for sensitivity analyses.

**Appendix S1**. Estimation of vaccine effectiveness for the prevalence of HPV.

Supplementary Material

Supplementary Material

Supplementary Material

Supplementary Material

Supplementary Material

Supplementary Material

Supplementary Material

## Data Availability

All data used for the analysis were derived from publicly available resources. We used national statistics and previously published estimates in order to estimate the number of women attending colposcopy. All this information is provided in the manuscript and there are no additional data to be shared.

## Legends for Figure 1

Example 1 for unvaccinated cohorts in 2018. (*)

- Numbers of women in each birth cohort: population forecast, retrieved from the Office for National Statistics^5^ and decreased by age‐specific prevalence of hysterectomy.^7^
- Numbers of women screened estimated as follows: (A) × coverage rate.^4^
- Numbers of women with a colposcopy after the baseline screening test:
  - For 46% of women screened with cytology: (B) × proportion referred to colposcopy in cytology screening × adherence with colposcopy referral × 0.46
  - For 54% of women screened with HPV testing: (B) × proportion referred to colposcopy at baseline in HPV‐based screening × adherence with colposcopy referral × 0.54.
- Numbers of women with a colposcopy after the 12‐month early recall in HPV screening (in 2019, for women screened in 2018): (B) × proportion with a colposcopy after the 12‐month early recall × 54%.
- Numbers of women with a colposcopy after the 24‐month early recall in HPV screening (in 2020, for women screened in 2018): (B) × proportion with a colposcopy after the 24‐month early recall × 54%.
- Numbers of CIN2+:
  - For women screened with cytology: (C[a]) × positive predictive value for CIN2+ at baseline in cytology screening × 46%.
  - For women screened with HPV testing:
    - (C[b]) × positive predictive value for CIN2+ at baseline in HPV‐based screening × 54%.
    - Number of women with CIN2+ diagnosis (in 2019, for women screened in 2018): (D) × positive predictive value for CIN2+ at 12‐month early recall in HPV‐based screening × 54%.
    - Number of women with CIN2+ diagnosis (in 2020, for women screened in 2018): (E) × positive predictive value for CIN2+ at 24‐month early recall in HPV‐based screening × 54%.

Example 2 for unvaccinated cohorts after 2018. (**)

- As in Example 1, for the subsequent calendar years.
- As in Example 1.
- Number of women with a colposcopy after the baseline screening test: (B) × proportion referred to colposcopy at baseline in HPV screening × adherence to colposcopy.
- Number of women with a colposcopy after the 12‐month early recall: (B) × proportion with a colposcopy after the 12‐month early recall.
- Number of women with a colposcopy after the 24‐month early recall: (B) × proportion with a colposcopy after the 24‐month early recall.
- Number of CIN2+:
  - (C) × positive predictive value for CIN2+ at baseline in HPV‐based screening.
  - (D) × positive predictive value for CIN2+ at 12‐month early recall in HPV‐based screening.
  - (E) × positive predictive value for CIN2+ at 24‐month early recall in HPV‐based screening.

Notes

‐ In calendar years 2018 and 2019, the effect of the HPV pilot from 2017 was considered by accounting for 12‐ and 24‐month early recall colposcopies, respectively, for 20% of the screened population targeted during three‐quarters of the year 2017.
‐ Estimates were applied for the first or the subsequent HPV screening rounds, as relevant.
‐ For vaccinated cohorts, all estimates from Table 2 were corrected for vaccine effectiveness (see text and Supplementary information)
