# Supplementary material for: Introducing human papillomavirus (HPV) primary testing in the age of HPV vaccination: projected impact on colposcopy services in Wales
Source: BJOG. 2020 Dec 15;128(7):1226–35. doi: 10.1111/1471-0528.16610 (PMC8246959; doi:10.1111/1471-0528.16610)
Supplement: Supplementary file 2 — Figure S2. Sensitivity analyses for the number of women with a CIN2+ diagnosis, under the current screening interval (A) and 5‐yearly intervals (B). [file BJO-128-1226-s005.pdf]

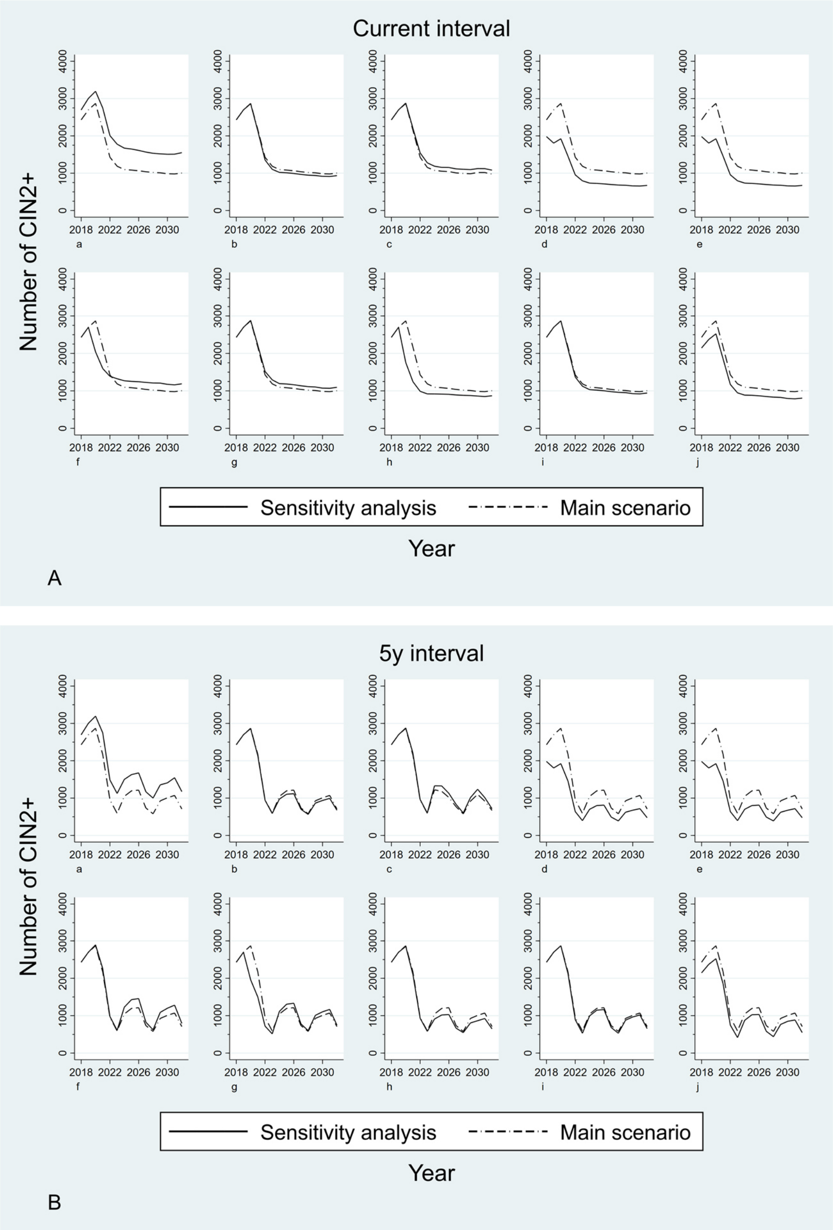

Figure S2. Sensitivity analyses for the number of women with a CIN2+ diagnosis, under the current screening interval (panel A) and 5-yearly intervals (panel B).

Scenarios:

a No Vaccination.

b Halved proportion of first HPV screens in the subsequent HPV rounds

c Doubled proportion of first HPV screens in the subsequent rounds

d Proportion with colposcopy decreased by one-third

e Proportion with colposcopy increased by one-third

f 50% more colposcopies in the subsequent HPV round

g PPV for CIN2+ in the subsequent HPV rounds equal those in the first HPV round

h PPV for CIN2+ in the subsequent HPV rounds halved compared to those in the first HPV round

i Vaccine effectiveness increased for herd immunity

j Vaccine effectiveness as in Scottish studies
