# Supplementary figures and images for: Introducing human papillomavirus (HPV) primary testing in the age of HPV vaccination: projected impact on colposcopy services in Wales
Source: BJOG. 2020 Dec 15;128(7):1226–35. doi: 10.1111/1471-0528.16610 (PMC8246959; doi:10.1111/1471-0528.16610)

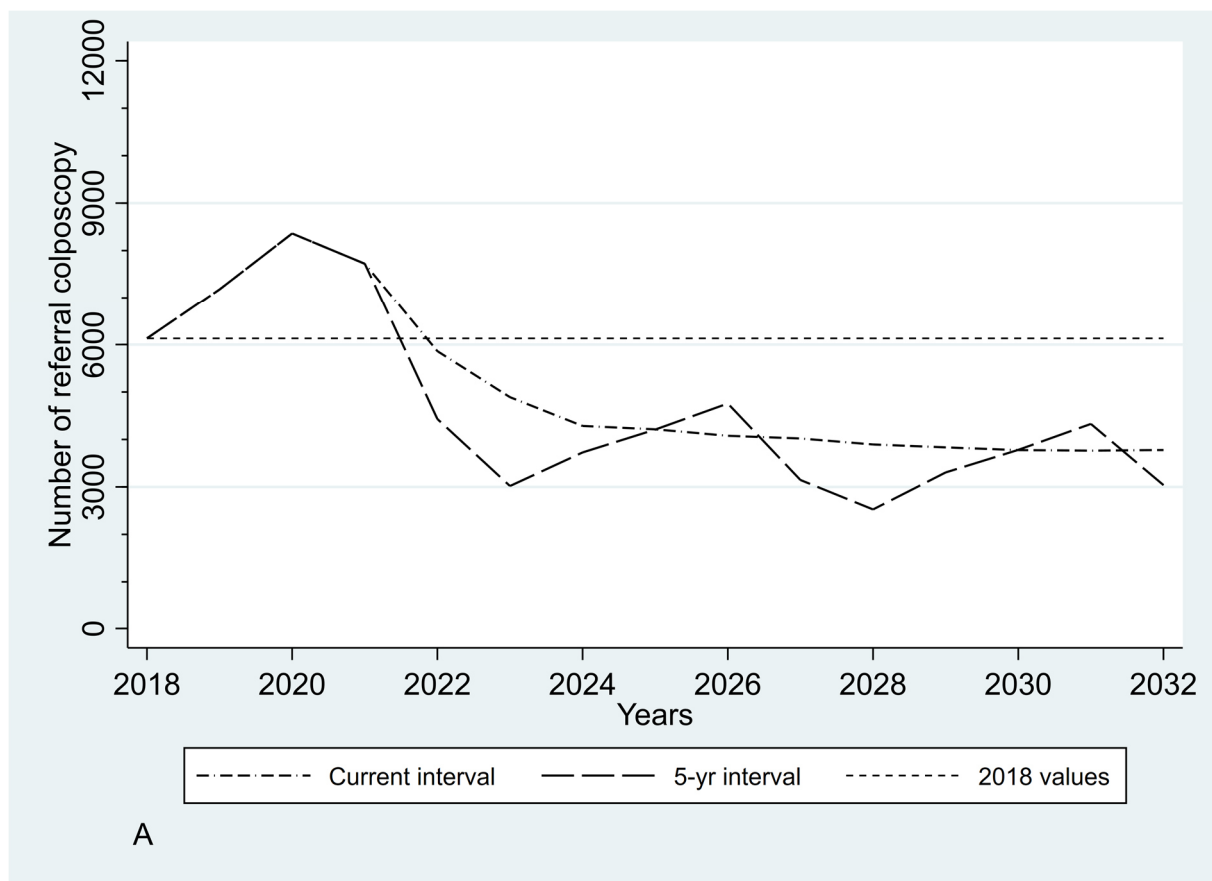

**Figure S3.** Panel A of Figure 2: numbers of women undergoing a screening-related colposcopy.

Supplement: Supplementary file 3 — Figure S3. Panel A of Figure 2: numbers of women undergoing a screening‐related colposcopy. [file BJO-128-1226-s008.pdf]
