# Supplementary material for: Introducing human papillomavirus (HPV) primary testing in the age of HPV vaccination: projected impact on colposcopy services in Wales
Source: BJOG. 2020 Dec 15;128(7):1226–35. doi: 10.1111/1471-0528.16610 (PMC8246959; doi:10.1111/1471-0528.16610)
Supplement: Supplementary file 4 — Figure S4. Panel B of Figure 2: total numbers of women undergoing colposcopy (i.e. screening‐related or clinical colposcopy). [file BJO-128-1226-s001.pdf]

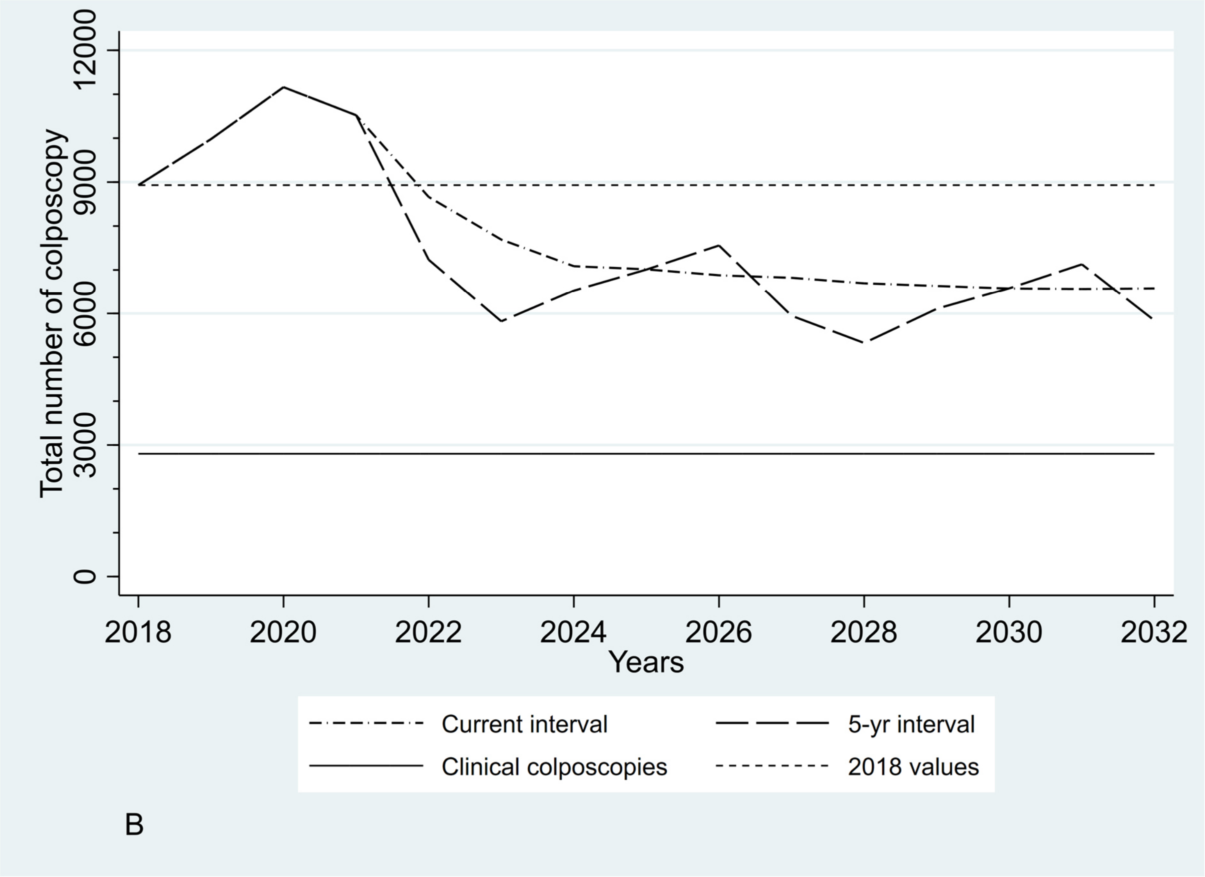

Figure S4. Panel B of Figure 2: total numbers of women undergoing colposcopy (i.e. screening-related or clinical colposcopy).
