# Supplementary material for: Introducing human papillomavirus (HPV) primary testing in the age of HPV vaccination: projected impact on colposcopy services in Wales
Source: BJOG. 2020 Dec 15;128(7):1226–35. doi: 10.1111/1471-0528.16610 (PMC8246959; doi:10.1111/1471-0528.16610)
Supplement: Supplementary file 5 — Figure S5. Panel C of Figure 2: numbers of women with a CIN2+ diagnosis after a screening referral, by screening scenario. [file BJO-128-1226-s015.pdf]

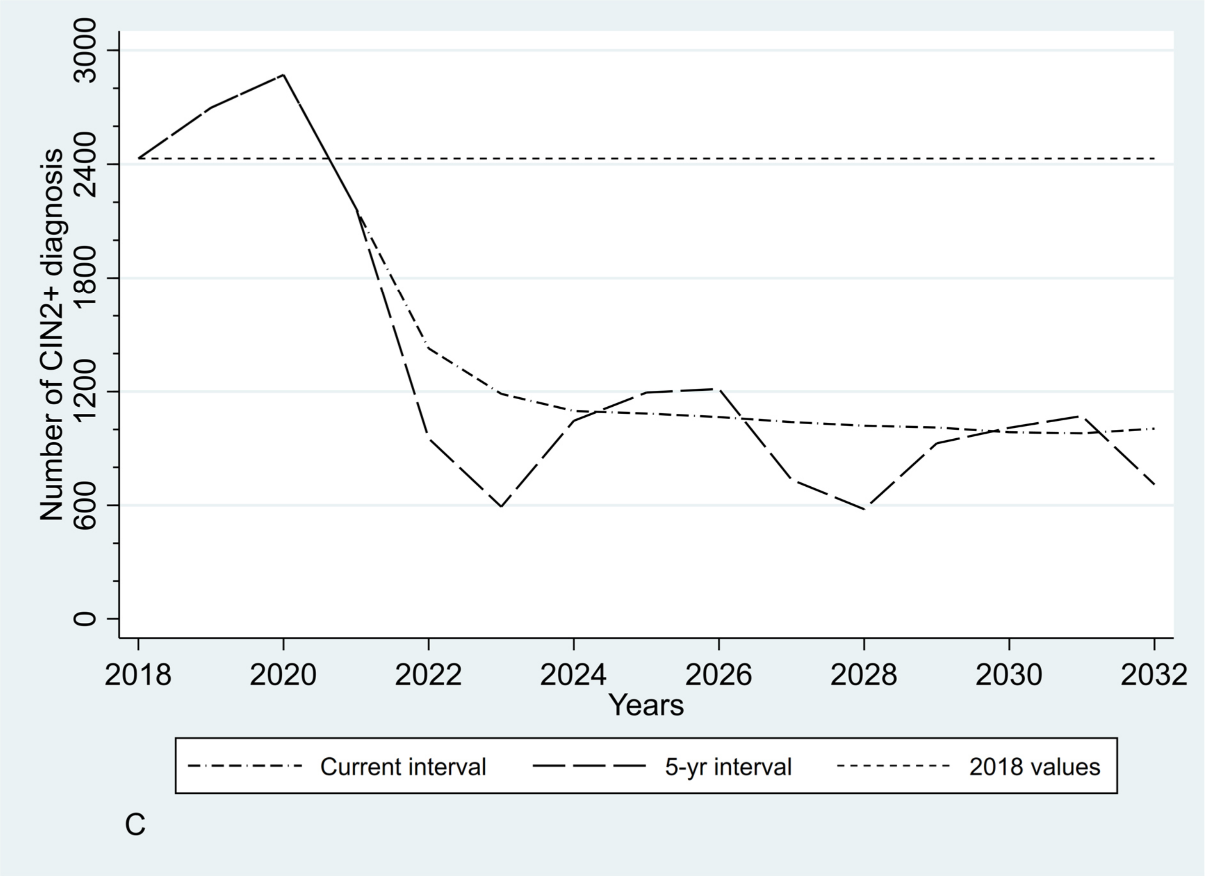

Figure S5. Panel C of Figure 2: numbers of women with a CIN2+ diagnosis after a screening referral, by screening scenario.
