# Supplementary material for: Introducing human papillomavirus (HPV) primary testing in the age of HPV vaccination: projected impact on colposcopy services in Wales
Source: BJOG. 2020 Dec 15;128(7):1226–35. doi: 10.1111/1471-0528.16610 (PMC8246959; doi:10.1111/1471-0528.16610)
Supplement: Supplementary file 6 — Figure S6. Panel D of Figure 2: number of women undergoing a screening‐related colposcopy estimated under the no vaccination scenario. [file BJO-128-1226-s003.pdf]

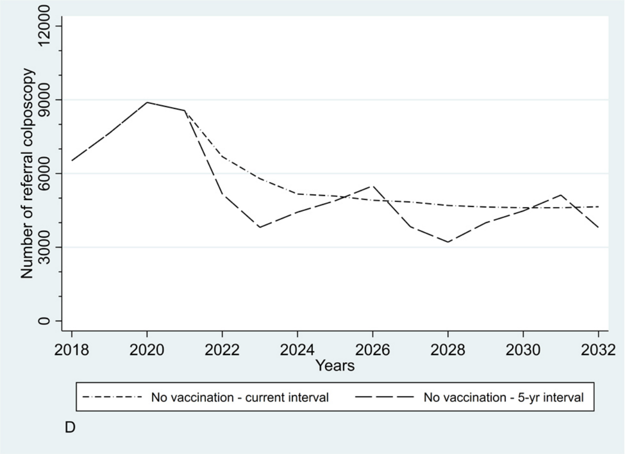

Figure S6. Panel D of Figure 2: number of women undergoing a screening-related colposcopy estimated under the no vaccination scenario.
