# Supplementary material for: Introducing human papillomavirus (HPV) primary testing in the age of HPV vaccination: projected impact on colposcopy services in Wales
Source: BJOG. 2020 Dec 15;128(7):1226–35. doi: 10.1111/1471-0528.16610 (PMC8246959; doi:10.1111/1471-0528.16610)
Supplement: Supplementary file 7 — Table S1. HPV vaccination coverage in Wales, by birth cohort. Data reported in COVER reports. Table S2. List of parameters used for sensitivity analyses. [file BJO-128-1226-s006.pdf]

Table S1. HPV vaccination coverage in Wales, by birth cohort. Data reported in COVER reports[11]

Birth cohort[a]	Vaccination cohort	Age at vaccination	Vaccination coverage				Targeted for screening since year
			3 doses (fully vaccinated)	2 doses (fully vaccinated)	1 dose (at least minimally protected)	COVER report Number	
1990-1991	Catch-up	17-18	30.2%	NR	47.8%	94	2015-16
1991-1992	Catch-up	17-18	34.6%	NR	49.7%	96	2016-17
1992-1993	Catch-up	16-17	49.3%	NR	62.4%	99	2017-18
1993-1994	Catch-up	15-16	72.0%	NR	81.6%	100	2018-19
1994-1995	Catch-up	14-15	79.6%	NR	84.0%	104	2019-20
1995-1996	Routine	12-13	87.2%	NR	89.9%	108	2020-21
1996-1997	Routine	12-13	83.9%	NR	86.6%	111	2021-22
1997-1998	Routine	12-13	86.8%	NR	88.3%	116	2022-23
1998-1999	Routine	12-13	87.0%	NR	89.4%	118	2023-24
1999-2000	Routine	12-13	86.8%	NR	89.9%	118	2024-25
2000-2001	Routine	12-13	84.8%	NR	90.5%	120	2025-26
2001-2002	Routine	12-13	NR	87.2%	91.3%	124	2026-27
2002-2003	Routine	12-13	NR	84.7%	89.0%	128	2027-28
2003-2004	Routine	12-13	NR	80.3%	89.3%	128	2028-29
2004-2005	Routine	12-13	NR	NA	85.6%	128	2029-30

Abbreviations: NA=not available. NR=not relevant.

[a] Girls born between September in year 1 and August in year 2. For girls born between September 1990 and August 2001, three vaccine doses were recommended, whereas girls born from September 2001 onwards receive two doses.

Note: Data for women born after 2004-05 have not yet been reported. For these birth cohorts, we assumed that the vaccine coverage rates will remain stable compared to the preceding routinely vaccinated birth cohorts.

Table S2. List of parameters used for sensitivity analyses

Parameter	Value for sensitivity analysis	Scenario in Figures S1 and S2
No vaccination	Vaccine effectiveness = 0	A
Main analysis	--	
Halved proportion of first HPV screens in the subsequent HPV rounds	5%	B
Doubled proportion of first HPV screens in the subsequent rounds	20%	C
Proportion with colposcopy decreased by one-third	Any colposcopy frequency * 0.67	D
Proportion with colposcopy increased by one-third	Any colposcopy frequency * 1.33	E
50% more colposcopies in the subsequent HPV round	Subsequent round colposcopy frequency * 1.5	F
PPV for CIN2+ in the subsequent HPV rounds equal those in the first HPV round	Same as PPVs for CIN2+ during the first HPV round	G
PPV for CIN2+ in the subsequent HPV rounds halved compared to those in the first HPV round	First-round PPVs for CIN2+ * 0.5	H
Vaccine effectiveness increased for herd immunity	For routinely vaccinated women: decrease in colposcopy 38% for bivalent and 29% for quadrivalent vaccine; decrease in the risk of CIN2+ 64% for bivalent and 49% for quadrivalent vaccine	I
Vaccine effectiveness as in Scottish studies	For routinely vaccinated women: decrease in colposcopy 43% for bivalent and 33% for quadrivalent vaccine; decrease in the risk of CIN2+ 77% for bivalent and 59% for quadrivalent vaccine<br><br>Women vaccinated through the catch-up campaign: decrease in colposcopy 17%; decrease in the risk of CIN2+: 39%	J
