# Supplementary material for: Introducing human papillomavirus (HPV) primary testing in the age of HPV vaccination: projected impact on colposcopy services in Wales
Source: BJOG. 2020 Dec 15;128(7):1226–35. doi: 10.1111/1471-0528.16610 (PMC8246959; doi:10.1111/1471-0528.16610)
Supplement: Supplementary file 8 — Appendix S1. Estimation of vaccine effectiveness for the prevalence of HPV. [file BJO-128-1226-s013.pdf]

**Appendix S1.** Estimation of vaccine effectiveness for the prevalence of HPV.

HPV genotype distribution in an unvaccinated population was estimated from the ARTISTIC trial.[1,2] The total proportions of unvaccinated women with HPV 16/18, HPV 31/33/45, and other high-risk HPV infections not including HPV 16/18/31/33/45 (including single and multiple infections) were estimated by comparing the reported numbers with genotype-specific single infections (detected in 7.8% of screened women) and the total reported proportion of women with any high-risk HPV infection detected by the clinically validated screening assay Hybrid Capture 2 (15.4%). To estimate the decrease in HPV positivity in vaccinated women, we applied effectiveness estimates for the bivalent vaccine from a recent English study.[3] That study estimated vaccine effectiveness against HPV 16/18 as 48.7% in the catch-up and 82.0% in the routinely vaccinated cohort (both reported estimates were statistically significant); and effectiveness against HPV 31/33/45 as 36.7% and 54.3%, respectively (the estimate for the catch-up cohort was not statistically significant). Although some vaccine effectiveness was reported for HPV 31/33/45/52/58, the estimates were smaller and not statistically significant, and we assumed no effectiveness of the bivalent vaccine against non-HPV 16/18/31/33/45 genotypes. According to these estimates and assuming that all girls were vaccinated, the proportion of women with any high-risk HPV infection would decrease from 15% in an unvaccinated population to 12% in the catch-up vaccinated population (a decrease of 21%), and to 10% in the routinely vaccinated population (a decrease of 35%). Taking into account the vaccine coverage rates (ca. 55% in the catch-up and 85% in the routine vaccination cohorts), we estimated that the population-based decrease in HPV positivity would be closer to 12% and 29%, respectively, which were our estimates of bivalent vaccine effectiveness against any high-risk HPV in a screening setting.

We applied quadrivalent or bivalent vaccine effectiveness estimates from the FUTURE I/II and PATRICIA randomised trials, respectively, against genotype-specific persistent HPV infection (or, if not available, seroconversion by the end of the study)[4-7] to the estimated genotype-specific prevalence in an unvaccinated ARTISTIC population.[1,2] This suggested that the effectiveness of the quadrivalent vaccine in a routinely vaccinated population is about 76% of that which could be expected with a bivalent vaccine. Hence, we assumed that the

effect on the overall HPV positivity in a birth cohort routinely vaccinated with a quadrivalent vaccine would be 22% (compared to 29% in birth cohorts routinely vaccinated with a bivalent vaccine).

In Wales, the APTIMA HPV assay is used for screening and HPV positive women are triaged to colposcopy following abnormal cytology. Scottish data showed that when screening samples were tested with the APTIMA assay, the reduction in HPV positivity was 32% for vaccinated vs. unvaccinated women, and the reduction in HPV positivity combined with abnormal cytology (a proxy for colposcopy referral) was 37%. This means that the reduction in the number of women referred for a colposcopy was about 15% (37/32) stronger than the reduction in the number of HPV positive women (see above for the estimation of the reduction in HPV positivity).[8]

In the PATRICIA trial of HPV vaccination, the bivalent vaccine effectiveness for CIN2+ in the youngest age group (15-17 years at vaccination and naïve to HPV infection) was 67.8% compared to unvaccinated controls.[9] The effectiveness of the bivalent vaccine in the catch-up population was about half that in the routinely vaccinated population in a recent English study.[3] As with the observed relative protection against persistent HPV infections in routinely vaccinated women (76%, see above), we assumed that the effectiveness of the quadrivalent vaccine against CIN2+ is 24% less than that for the bivalent vaccine (due to the greater cross-protection of Cervarix).


1. Sargent A, Bailey A, Almonte M, Turner A, Thomson C, Peto J *et al.* Prevalence of type-specific HPV infection by age and grade of cervical cytology: data from the ARTISTIC trial. *British journal of cancer* 2008; **98**(10): 1704-1709; e-pub ahead of print 2008/04/09; doi 10.1038/sj.bjc.6604324.
2. Kitchener HC, Almonte M, Gilham C, Dowie R, Stoykova B, Sargent A *et al.* ARTISTIC: a randomised trial of human papillomavirus (HPV) testing in primary cervical screening. *Health technology assessment (Winchester, England)* 2009; **13**(51): 1-150, iii-iv; e-pub ahead of print 2009/11/07; doi 10.3310/hta13510.
3. Mesher D, Panwar K, Thomas SL, Edmundson C, Choi YH, Beddows S *et al.* The Impact of the National HPV Vaccination Program in England Using the Bivalent HPV Vaccine: Surveillance of Type-Specific HPV in Young Females, 2010-2016. *The Journal*

*of infectious diseases* 2018; **218**(6): 911-921; e-pub ahead of print 2018/06/20; doi 10.1093/infdis/jiy249.

4. Wheeler CM, Castellsague X, Garland SM, Szarewski A, Paavonen J, Naud P *et al.* Cross-protective efficacy of HPV-16/18 AS04-adjuvanted vaccine against cervical infection and precancer caused by non-vaccine oncogenic HPV types: 4-year end-of-study analysis of the randomised, double-blind PATRICIA trial. *The Lancet Oncology* 2012; **13**(1): 100-110; e-pub ahead of print 2011/11/15; doi 10.1016/s1470-2045(11)70287-x.
5. Lehtinen M, Paavonen J, Wheeler CM, Jaisamrarn U, Garland SM, Castellsague X *et al.* Overall efficacy of HPV-16/18 AS04-adjuvanted vaccine against grade 3 or greater cervical intraepithelial neoplasia: 4-year end-of-study analysis of the randomised, double-blind PATRICIA trial. *The Lancet Oncology* 2012; **13**(1): 89-99; e-pub ahead of print 2011/11/15; doi 10.1016/s1470-2045(11)70286-8.
6. Brown DR, Kjaer SK, Sigurdsson K, Iversen OE, Hernandez-Avila M, Wheeler CM *et al.* The impact of quadrivalent human papillomavirus (HPV; types 6, 11, 16, and 18) L1 virus-like particle vaccine on infection and disease due to oncogenic nonvaccine HPV types in generally HPV-naive women aged 16-26 years. *The Journal of infectious diseases* 2009; **199**(7): 926-935; e-pub ahead of print 2009/02/25; doi 10.1086/597307.
7. Joura EA, Kjaer SK, Wheeler CM, Sigurdsson K, Iversen OE, Hernandez-Avila M *et al.* HPV antibody levels and clinical efficacy following administration of a prophylactic quadrivalent HPV vaccine. *Vaccine* 2008; **26**(52): 6844-6851; e-pub ahead of print 2008/10/22; doi 10.1016/j.vaccine.2008.09.073.
8. Bhatia R, Kavanagh K, Cubie HA, Serrano I, Wennington H, Hopkins M *et al.* Use of HPV testing for cervical screening in vaccinated women--Insights from the SHEVa (Scottish HPV Prevalence in Vaccinated Women) study. *International journal of cancer* 2016; **138**(12): 2922-2931; e-pub ahead of print 2016/02/05; doi 10.1002/ijc.30030.
9. Apter D, Wheeler CM, Paavonen J, Castellsague X, Garland SM, Skinner SR *et al.* Efficacy of human papillomavirus 16 and 18 (HPV-16/18) AS04-adjuvanted vaccine against cervical infection and precancer in young women: final event-driven analysis of the randomized, double-blind PATRICIA trial. *Clinical and vaccine immunology : CVI* 2015; **22**(4): 361-373; e-pub ahead of print 2015/02/06; doi 10.1128/cvi.00591-14.
